# Effect of strontium ranelate and cerium oxide addition in the diet on bone quality and expression level of osteocalcin and alkaline phosphatase genes in broiler chicken

**DOI:** 10.1002/vms3.1190

**Published:** 2023-06-13

**Authors:** Payam Sadq Sabir, Kamaran A. Abbas

**Affiliations:** ^1^ Department of Animal Resource College of Agriculture Engineering Sciences Salahaddin University‐Erbil Erbil Iraq

**Keywords:** bone, broiler, cerium oxide, gene expression, strontium ranelate

## Abstract

**Background:**

In the modern broiler industry, leg and gait disorders are considerable problems. Fast‐growing broilers are especially susceptible to bone abnormalities, causing major problems for broiler producers. Strontium ranelate (SrR) has been used successfully for the treatment of osteoporosis in humans. In addition, cerium oxide (CeO) is an anti‐stress agent applied in the biological system.

**Methods:**

This study was conducted to investigate the effect of SrR, CeO, and their combinations on tibia quality in broilers. A total of 384 one‐day‐old Ross chicks were divided into six treatments, with four replicates per treatment (16 birds per replicate). The control group was fed a standard diet, and other groups were fed SrR at levels 450, 900 mg/kg feed, CeO at levels 300 and 600 mg/kg feed and a combination of 450 SrR + 300 CeO mg/kg feed. Bone mineral density (BMD), bone mineral content (BMC), bone strength (BS), tibia area, tibia weight, bone Length, bone diameter, minerals in tibia bone of male broilers, alkaline phosphatase gene (ALP) and osteocalcin gene (OC) in male broilers were analysed.

**Results:**

The results showed that the addition of SrR and CeO had no significant influence (*p* > 0.01) on BMD, BMC, BS, bone weight, bone length and bone diameter. While there was a significant interaction between sex and treatments, especially in the combination group, BS in females significantly (*p <* 0.01) increased compared to the control group. Generally, females were found to be more responsive to treatments than males. Significant increases in gene expression were noticed in OC with the addition of low levels of SrR and CeO and mixed group compared to the control. The gene expression of ALP was increased significantly only in a combination group compared to the control group.

**Conclusion:**

It is concluded that SrR and CeO can be used as beneficial additives in the feed to improve the tibia quality of broilers.

## Graphical abstract

This study indicates that the combination of SrR and CeO in the females significantly increased bone strength compared with the control group. Also, Sr can replace Ca in the mineral content of the growing tibia.

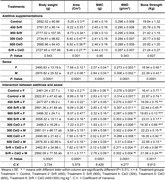

## 1 INTRODUCTION

Modern broilers grow three to four times as quickly as earlier generations (Browning & Cowieson, 2013). Leg disorders, bone weakness, and broken bones are common problems in broilers that are attributed to poor growth, downgrading at slaughter, and reducing the bird's routine activities, such as walking, standing, eating, and drinking subsequently causing economic loss at the end (Bell & Weaver, 2002; Manohar et al., 2015; Meseret, 2016; Kierończyk et al., 2017).

Lameness affects up to 2% of broiler flocks, costing the industry around $4 billion globally. Also, osteoporosis represents 6% of broilers’ abnormally developed skeletal or musculoskeletal disease (Ammann, 2006; Baracho et al., 2018, 2019; Bishop et al., 2000).

The dietary elements have the greatest impact on poultry bone strength (BS) (Adu & Olarotimi, 2020; Ammann, 2006; Bell & Weaver, 2002; Thorp et al., 1991). Although several additional inorganic elements are present in the bone that may be crucial for bone health and strength, calcium and phosphor are major inorganic nutrients because they make up 95% of the mineral matrices (Council, 1994). To promote BS in chickens, researchers have traditionally focused on calcium and phosphorus minerals and vitamin D (Fleming et al., 1998).

Inorganic (mineral) ingredients in bone tissue determine bone density and mechanical strength, whereas organic components ensure bone flexibility (Council, 1994). Bone mineral density (BMD) is a biophysical metric that is used to assess bone structure (Urist & Deutsch, 1960). A pathological mineralization deficiency is responsible for the decline in bone density that contributes to osteoporosis (Ensrud & Crandall, 2017).

Strontium (Sr) is an alkaline element and rare earth metal (Browning & Cowieson, 2013). The physical and chemical properties are similar to that of calcium (Skoryna, 1981). In addition, stable Sr isotopes do not have any significant health threat (Gulson & Wong, 2006). Sr functions as a moderately effective antiresorptive agent in humans (Tamba & Alexa‐Stratulat, 2017). In the clinical setting, therapeutic uses of Sr salts such as strontium ranelate (SrR) are involved in preventing osteoporotic fractures (Meunier et al., 2004). Sr appeared to stimulate bone formation and inhibit bone resorption in rodents (Tamba & Alexa‐Stratulat, 2017). In poultry, the average intake of Sr is about 19 mg/kg of feed (Doberenz et al., 1969).

SrR consists of two strontium atoms coupled by ranelic acid. It is used to treat osteoporosis (Pilmane et al., 2017). SrR improved bone mass/quality and increased BS in osteoporotic patients through changes in bone matrix characteristics and BMD in clinical trials (Bakhit et al., 2018). In addition, the use of SrR is approved in the United States, Europe and Australia to stimulate bone formation in humans (Suzuki et al., 2008).

Cerium oxide (CeO) is an oxide of the rare‐earth element consisting of the metal cerium with the chemical formula CeO_2_. CeO has many uses either commercially or biologically, and its impact on animals and poultry is studied by several workers (Adu & Olarotimi, 2020; Bölükbaşı et al., 2016; Rajeshkumar 2017; Reka et al., 2019). Numerous studies showed that Ce, as nanoparticles, gave a potential therapeutic tool in preventing and treating oxidative stress in animals and poultry (CórdobaJover 2019). In biological contexts, it has been reported that CeO nanoparticles can mimic enzymatic antioxidants such as superoxide dismutase (Heckert et al., 2008).

The distribution of enzymes and proteins involved in bone production and resorption can influence the process of bone remodelling. Furthermore, osteoporosis is caused by an imbalance in bone growth and resorption (Ahmadi & Ashrafizadeh, 2018). Various scientific evidence indicated the role of nutrition on specific gene expression, among those genes responsible for bone formation, alkaline phosphatase (ALP) and osteocalcin (OC) genes. ALP is an important component in the creation of hard tissue and is strongly expressed in mineralized tissue cells (Golub et al., 2007), which is involved in bone mineralization (Nelson et al., 2020). In addition, OC (bone γ‐carboxyglutamic acid protein) regulates bone metabolism, in which a decrease in the amount of the OC gene diminishes bone metabolism and predisposes bone to diseases (Ahmadi & Ashrafizadeh, 2018), OC is required for BS in the longitudinal direction of the long bone by regulating the alignment of biological apatite parallel to collagen fibrils and plays an important function in bone structure (Komori, 2020).

The objectives of this study were to examine the effect of SrR and CeO supplementations in the diet on the bone quality of broilers to improve the welfare of birds.

## 2 MATERIALS AND METHODS

### 2.1 Animals and sampling for analysing

This experiment was conducted in the poultry research hall at the College of Agriculture Engineering Science. A total of 384 one‐day‐old Ross 308 chicks were equally divided into six groups. In the first week of bird's age, all broilers were fed the basal control starter diet; then each period of dietary treatment starter, grower and finisher are shown in Table 1. The dietary treatments include a control diet, control diet supplemented with 450 and 900 mg/kg of SrR in second and third groups, respectively, and 300 and 600 mg/kg of CeO in fourth and fifth groups, respectively, and the final treatment was supplemented with a combination of 450 + 300 mg/kg of SrR and CeO. Each dietary treatment consisted of four replicates of 16 birds (eight males and eight females in each replicate). Feed and water were offered ad libitum throughout the experiment. When birds were 43 days old, 48 birds (one male and one female from each replicate), which represent the average body weight of each sex in the treatment selected and weighed individually, were slaughtered. On the same day, the right fresh leg was scanned using the dual‐energy X‐ray absorptiometry method (DEXA Scan, Siemens, 2000 Germany). For whole tibia bone samples, BMD (g/cm^2^) and bone mineral content (BMC; grams) were assessed. Then the samples were stored in the refrigerator for 1 week; the tibia was immersed in boiling water (95°C) for 12 min. The tibias were de‐fleshed, and the patella was removed. The samples were air‐dried for 24 h at room temperature. The BS was recorded by the hardness tester (TSS QC‐SPA, UK). Calcium, strontium, magnesium, and phosphorus as minerals were measured with the help of a wavelength dispersive X‐ray fluorescence spectrometer (S8 Tiger, Bruker, Germany).

**TABLE 1:** Ingredients (kg/100 kg) and nutrient composition of the experimental diets.

Ingredients	Starter diet (8–12 days)	Grower diet (13–25 days)	Finisher diet (26–43 days)
Soybean meal 46% protein	35.155	30.300	26.00
Corn	48.750	23.000	31.00
Wheat flour	10.000	38.580	36.470
Wheat bran	–	1.500	–
Premix^a^	1.000	1.000	0.900
l‐threonine	0.170	0.070	0.040
Limestone	1.660	1.500	1.390
Soybean oil	1.000	2.200	2.600
Lysine	0.420	0.260	0.290
Methionine	0.260	0.200	0.170
Sodium bicarbonate	0.540	0.400	0.380
Mono calcium phosphate	0.650	0.510	0.390
Table salt	0.170	0.210	0.170
Toxin binder	0.150	0.150	0.150
Enzyme^b^	0.050	0.050	0.050
lysofort^c^	0.025	0.020	–
Anti‐coccidian^d^	‐	0.050	–
Total	100	100	100
**Calculated value**			
Crude protein %	21.840	20.840	18.930
Metabolizable energy (kcal/kg)	2970	3000	3100
Methionine %	0.557	0.474	0.430
Lysine %	1.392	1.171	1.082
Methionine + cysteine %	0.833	0.755	0.692
Threonine %	0.933	0.782	0.683
Tryptophan %	0.239	0.236	0.210
Ash %	6.407	5.800	5.286
Fiber %	2.242	2.169	2.000
Ether extract %	3.737	4.412	2.361
Calcium %	0.960	0.873	0.782
Available phosphor %	0.480	0.436	0.390

^a^ The vitamins and trace minerals premix added according to *Ross 308 Broilers Requirements Guide* (2012); arginine, 3.5%; threonine, 6%; tryptophane, 0.5%; BHT, 830 mg/kg; propyl gallate, 70 mg/kg; citric acid, 125 mg/kg; betaine hydrochloride, 6000 mg/kg; crude protein: 13.4%, M.E: 360 kcal/kg, Ca: 30.7%, vitamins (A: 1,300,000 IU, D_3_: 500,000 IU, E: 8000 mg, K_3_: 320 mg, B1: 320 mg, B2: 860, B6: 540, B12: 1.7 mg, H: 30 mg), niacin: 6000 mg, folic acid: 220 mg, d‐pantothenic acid: 2000 mg, coline Chloride: 17000 mg, betaine: 6000 mg, Cu: 1600 mg, Mn: 12,000 mg, Zn: 6000, Fe: 2000 mg, I: 125 mg, Se: 30 mg. ^b^Kimzyme. ^c^Emulsifier. ^d^Sscox (salinomycin sodium).

### 2.2 Slaughtering procedure

The work was conducted by slaughtering 24 male and 24 female broilers in the Animal Resources Departmental Research Slaughter, College of Agriculture Engineering Sciences. The slaughtering was conducted in a research abattoir at the Department of Animal Resources College of Agriculture Engineering Sciences.

### 2.3 Gene expression measurement

#### 2.3.1 Primer design

Each of the forward and reverse primers was designed by the National Center for Biotechnology Information (NCBI) bioinformatics programme. Targeted genes are partial genes of *Gallus gallus* ALP and *Gallus gallus* OC, which were selected from the NCBI gene bank (https://www.ncbi.nlm.nih.gov/nucleotide) primers picked from the online programme of https://www.ncbi.nlm.nih.gov/tools/primer‐blast/primertool.cgi. After adding sequences of partial genes of ALP and OC clicked on picked primers so several forward and revere primers were produced (Table 2). The primers were synthesized in the Micro‐Gene of South Korea.

**TABLE 2:** Gene bank reference accession number.

Name of gene	Primer		Gene size (bp)	References
Beta‐actin	Forward	ATGGCTCCGGTATGTGCAA	150	X00182.1.
	Reverse	TGTCTTTCTGGCCCATACCAA		
ALP	Forward	ACCTACAACACCAACGCACA	184	MN_205360
	Reverse	CCGCCTTGCCTTCATCCTTA		
OC	Forward	CTTCATCTCCCACCGCCA	126	U10578
	Reverse	CTCAGCTCACACACCTCTCG		

Abbreviations: ALP, alkaline phosphatase; OC, osteocalcin.

#### 2.3.2 RNA extraction and complementary DNA synthesis

At the end of the experiment, one male bird from each replicate was slaughtered and samples of bone marrow were taken from the tibia. RNA was extracted from fresh tibia bone marrow using an extraction kit for tissue obtained from Favorgene RNA (Korea).

Five micrograms of total RNA was converted to cDNA by the Beta Script Kit for cDNA synthesis from (Beta Bayern GMbH, Nurnberg, Germany) following the manufacturer's instructions.

#### 2.3.3 Real‐time polymerase chain reaction amplification

Polymerase chain reaction(PCR) amplification of genes was done in 20 μL of reaction mixture containing 2× SYBER Green Master(Addbio, Korea), 10 pmol of forwards, 10 pmol reverse primer, DNase‐free water, and a template of cDNA (Applied Bio‐system Model 7500 Real‐Time thermos‐cycler) was run. The temperature profile included the following steps: step one was an initial denaturation at 95°C for 5 min, step two followed by 40 cycles of denaturation at 95°C for 35 s, primer annealing at different temperatures according to primers of genes (59, 60, 61)°C for each of beta‐actin, OC, ALP, respectively, for 45 s, an extension at 72°C for 1 min and final step was an extra extension at 72°C for 5 min.

Each sample was run in duplicate, and averaged duplicates were used to assign cycle threshold (CT) values. The ΔCT values were generated by subtracting experimental CT values from the CT values for beta‐actin targets amplified with each sample. The average of the control group with the highest means ΔCT value (lowest gene expression) per amplified gene target was calculated, and the mean ΔCT values of the other groups were set relative to this calibrator (ΔΔCT). The ΔΔCT values were calculated as powers of (2*^−^* ^ΔΔCT^) to account for the exponential doubling of the PCR (Livak & Schmittgen, 2001). Then, the geometric mean was calculated for the (2*^−^* ^ΔΔCT^) of each replicate in the groups and then the standard (SE) error was calculated to draw the figure.

### 2.4 Statistical analysis

Statistical analysis of the data (bone area, BMC, BMD, BS, bone weight, bone length, and bone diameter) is presented as means ± SE. One‐way analysis of variance (ANOVA) followed by a factorial test to evaluate the effect of additives and sexes on treats, was performed using SAS program version 9.3. Differences were reported as significant in all instances with a *p* < 0.05. Statistical analysis of the data of tibia (mineral content) is presented as means ± SE. One‐way ANOVA followed by a Completely Randomized Design test in order to determine the effect of additives on treats was performed using SAS program version 9. Differences were reported as significant in all instances with a *p* < 0.05.

To evaluate the homogeneity of variances and normality of data, as well as to determine the significance and interactions of main effects, the SAS statistical software (PROC GLM) was utilized (SAS, 9.3, 2013). Duncan's multiple range test was employed to discover differences between individual treatment means and interactions were observed in *p* < 0.05.

## 3 RESULTS AND DISCUSSION

### 3.1 Body weight

The addition of SrR, CeO and their combination had no significant (*p* > 0.01) effect on body weight (Table 4).

### 3.2 Bone quality

Rapid growth in birds immediately post‐hatches imposes a high mechanical load on the skeleton, which is still underdeveloped (Yair et al., 2012). The impact of SrR, CeO, and their combination on bone quality is shown in Table 3. The results showed that no significant (*p* > 0.01) differences were observed among treatments, except the tibia area, if the group fed 900 mg/kg of SrR increased significantly (*p* < 0.01) compared with the group fed 450 mg/kg of SrR. While there was a significant (*p* < 0.01) difference in all parameters between males and females. On the other hand, there is a significant (*p* < 0.01) interaction between sex and treatments especially in females in the group fed 900 mg/kg SrR; the area of the tibia was significantly more than in females in the group fed 450 mg/kg SrR and females that fed 300 mg/kg CeO. This significant increase in the area related to the increase in the diameter of the tibia in this group is shown in Table 4; in addition to the increase in the cortical and medullary area of the tibia, the use of Sr resulted in an increase in the total area of the tibia (Shahnazari et al., 2007)

**TABLE 3:** Represents the effects of SrR, CeO and their combination on Bone quality.

Treatments	Body weight (g)	Area (cm^2^)	BMC (g)	BMD (g/cm^2^)	Bone strength (kg)
Additive supplementations					
Control	2692.02 ± 90.86	8.25 ± 0.3^ab^	2.46 ± 0.15	0.296 ± 0.008	19.64 ± 1.32
450 SrR	2761.57 ± 95.14	8.22 ± 0.33^b^	2.40 ± 0.16	0.290 ± 0.008	20.76 ± 0.76
900 SrR	2777.32 ± 107.05	8.73 ± 0.34^a^	2.58 ± 0.13	0.295 ± 0.004	21.42 ± 1.15
300 CeO	2734.61 ± 98.62	8.40 ± 0.47^ab^	2.41 ± 0.19	0.284 ± 0.007	20.77 ± 0.92
600 CeO	2688.96 ± 84.43	8.52 ± 0.38^ab^	2.48 ± 0.18	0.288 ± 0.009	20.92 ± 1.20
SrR + CeO	2727.45 ± 107.49	8.46 ± 0.27^ab^	2.44 ± 0.13	0.287 ± 0.007	21.24 ± 0.27
*p*‐value	0.543	0.001	0.48	0.343	0.627
Sexes					
F	2480.83 ± 13.19 b	7.58 ± 0.12^b^	2.07 ± 0.04^b^	0.273 ± 0.003^b^	18.94 ± 0.45^b^
M	2970.82 ± 25.52 a	9.27 ± 0.08^a^	2.84 ± 0.04^a^	0.306 ± 0.002^a^	22.39 ± 0.43^a^
*p*‐value	0.0001	0.0001	0.0001	0.0001	0.0001
Interaction between additives and sexes					
Control × F	2461.24 ± 27.7 c	7.52 ± 0.2^bc^	2.09 ± 0.06^bc^	0.278 ± 0.003^cd^	16.41 ± 0.77 ^d^
Control × M	2922.81 ± 47.42 ab	8.99 ± 0.18^a^	2.83 ± 0.12^a^	0.314 ± 0.007^a^	22.87 ± 0.74^a^
450 SrR × F	2477.87 ± 65.2 c	7.30 ± 0.22^c^	1.97 ± 0.05^c^	0.270 ± 0.007 ^cd^	19.09 ± 0.75 ^bcd^
450 SrR × M	2991.36 ± 41.84 ab	9.09 ± 0.26^a^	2.80 ± 0.18^a^	0.307 ± 0.011^a^	22.44 ± 0.45^ab^
900 SrR × F	2496.32 ± 24.7 c	8.07 ± 0.47^b^	2.32 ± 0.16^b^	0.287 ± 0.006^bc^	19.83 ± 0.13 ^abcd^
900 SrR × M	3058.32 ± 15.11 a	9.40 ± 0.16^a^	2.85 ± 0.08^a^	0.303 ± 0.004^ab^	23.01 ± 0.17^a^
300 CeO × F	2488.2 ± 16.26 c	7.22 ± 0.27^c^	1.93 ± 0.11^c^	0.266 ± 0.006 ^d^	19.30 ± 0.66 ^abcd^
300 CeO × M	2981.01 ± 68.17 ab	9.58 ± 0.15^a^	2.89 ± 0.08^a^	0.302 ± 0.004^ab^	22.23 ± 0.15^ab^
600 CeO × F	2498.56 ± 21.42 c	7.58 ± 0.18^bc^	2.02 ± 0.07^bc^	0.266 ± 0.008 ^d^	18.35 ± 0.92 ^cd^
600 CeO × M	2879.35 ± 92.95 b	9.47 ± 0.19^a^	2.94 ± 0.09^a^	0.31 ± 0.004^a^	22.85 ± 0.15^a^
SrR + CeO × F	2462.81 ± 34.86 c	7.82 ± 0.19^bc^	2.12 ± 0.08^bc^	0.271 ± 0.004 ^cd^	21.49 ± 0.56^abc^
SrR + CeO × M	2992.09 ± 77.55 ab	9.11 ± 0.17^a^	2.76 ± 0.09^a^	0.302 ± 0.004^ab^	21.06 ± 0.24^abc^
*p*‐value	0.0001	0.0001	0.0001	0.0001	0.0017
CV	3.734	5.579	8.429	4.277	9.813

*Note*: Means ± SE

Abbreviations: BMC, bone mineral content; BMD, bone mineral density; CeO, cerium oxide; CV, coefficient of variance; F, female; M, male; SrR, strontium ranelate.

^a,..d.^ means with different letters within a column are significantly different (*p* < 0.01). *n* = 4. Treatment groups: Treatment 1: Control, Treatment 2: SrR (450), Treatment 3: SrR (900), Treatment 4: CeO (300), Treatment 5: CeO (600), Treatment 6: SrR + CeO (450+300) mg/kg.

**TABLE 4:** Represents the effects of SrR, CeO and their combination on Bone measurements.

Treatments	Tibia weight (g)	Tibia length (mm)	Tibia diameter (mm)
Additive supplementations			
Control	7.38 ± 0.544	96.98 ± 1.636	9.00 ± 0.288
450 SrR	7.29 ± 0.431	97.70 ± 1.197	8.97 ± 0.242
900 SrR	7.49 ± 0.449	97.96 ± 1.758	8.99 ± 0.255
300 CeO	7.35 ± 0.541	98.56 ± 1.46	9.04 ± 0.373
600 CeO	7.56 ± 0.449	98.28 ± 0.817	8.97 ± 0.254
SrR + CeO	7.25 ± 0.512	98.20 ± 1.057	8.62 ± 0.339
*p* Value	0.854	0.813	0.687
Sexes			
Female	6.16 ± 0.101^b^	95.03 ± 0.515^b^	8.30 ± 0.112^b^
Male	8.59 ± 0.117^a^	100.80 ± 0.449^a^	9.55 ± 0.099^a^
*p*‐value	0.0001	0.0001	0.0001
Interaction between additives and sexes			
Control × F	6.05 ± 0.301^b^	93.85 ± 2.222^c^	8.32 ± 0.186^b^
Control × M	8.70 ± 0.344^a^	100.10 ± 1.018^ab^	9.69 ± 0.196^a^
450 SrR × F	6.05 ± 0.15^b^	94.59 ± 1.052^c^	8.28 ± 0.017^b^
450 SrR × M	8.43 ± 0.371^a^	100.44 ± 1.251^ab^	9.57 ± 0.249^a^
900 SrR × F	6.45 ± 0.263^b^	94.17 ± 1.292^c^	8.54 ± 0.371^b^
900 SrR × M	8.53 ± 0.392^a^	101.75 ± 1.778^a^	9.44 ± 0.171^a^
300 CeO × F	6.00 ± 0.286^b^	95.04 ± 0.788^c^	8.33 ± 0.400^b^
300 CeO × M	8.7 ± 0.261^a^	102.08 ± 1.035^a^	9.75 ± 0.392^a^
600 CeO × F	6.45 ± 0.253^b^	96.85 ± 1.118^bc^	8.44 ± 0.246^b^
600 CeO × M	8.68 ± 0.229^a^	99.71 ± 0.71^ab^	9.50 ± 0.226^a^
SrR + CeO × F	5.98 ± 0.246^b^	95.71 ± 0.767^c^	7.89 ± 0.319^b^
SrR + CeO × M	8.53 ± 0.278^a^	100.70 ± 0.697^ab^	9.36 ± 0.278^a^
*p* Value	0.0001	0.0001	0.0001
CV	7.812	2.501	6.162

*Note*: Means ± SE.

Abbreviations: CeO, cerium oxide; CV, coefficient of variance; F, female; M, male; SrR, strontium ranelate.

^a,..c.^ means with different letters within a column are significantly different (*p* < 0.01). *n* = 4. Treatment groups: Treatment 1: Control, Treatment 2: SrR (450), Treatment 3: SrR (900), Treatment 4: CeO (300), Treatment 5: CeO (600), Treatment 6: SrR + CeO (450 + 300) mg/kg.

The impact of additives on BMC in treatments was not significant. While in females, a significant (*p* < 0.01) increase was observed between a group fed 900 mg/kg SrR compared to groups fed 450 mg/kg SrR and 300 mg/kg CeO; this difference was related to increasing the area of the tibia. Marie et al. (1993) obtained a similar result with ovariectomized osteopenic rats when fed strontium salt. Additionally, Ammann et al. (2004) noted that Sr therapy increased BMC in female rats. Similarly, Shahnazari et al. (2007) reported that BMC increased significantly with Sr addition to the fed in broilers.

The addition of SrR, CeO and their combination had no significant (*p* > 0.01) impact on the BMD. While BMD in males is significantly (*p* < 0.01) higher than BMD in females as well as there is a significant (*p* < 0.01) interaction between treatments and sex in BMD of the tibia. BMD in females significantly (*p* < 0.01) increased in the group that received SrR (900 mg/kg) compared with both 300 mg/kg CeO and 600 mg/kg CeO. Aveline et al. (2021) found a significant increase in BMD of ovariectomized rats that received 625 mg/kg/day of SrR. Ammann et al. (2004) also revealed that Sr has a substantial impact on BMD. This variation with previous data may be due to the animal species used in the research.

The addition of SrR, CeO, and their combination has no significant effect on BS in treatments. The results showed a significant difference between males and females, BS in males was significantly (*p* < 0.01) higher than BS in females. While there was a significant (*p* < 0.01) interaction between treatments and sex. Sex is a factor that influences bone growth. Size differences, as well as hormonal differences, can account for the differences in growth and BS between males and females (Rath et al., 1999). In the combination group (450 SrR + 300 CeO mg/kg feed), females' BS increased significantly more than in the control group. That means dietary SrR and CeO supplementation has the ability to reduce lameness and osteoporosis in broiler chickens. Bone problems have been a key issue that perilously affects broilers' health and welfare, resulting in severe economic loss (Nakhon et al., 2019). Bone structural and metabolic disturbances (like tibial dyschondroplasia, osteoporosis, and osteoarthrosis) are common in broiler chickens (Khan et al., 2021). In a study on females, Meunier et al. (2009) revealed a significant increase in BS in the group that was administered Sr. Similarly, Ammann et al. (2004) stated that treated rats with 900 mg/kg/day had a stronger bone in comparison to the other groups. Also in a study with rats, when fed a diet containing 625 mg/kg/day, SrR showed anti‐fracture efficacy by influencing the determinants of BS (Bain et al., 2009). In addition, Ferraro et al. (1983), Dahl et al. (2001), Grynpas and Marie (1990), Marie et al. (2001), and Marie and Hott (1986) observed that supplementing (316‐634 mg/kg/day Sr^2+^) inhibited bone resorption by inhibiting the action of bone osteoclast cells, resulting in an increase in bone volume with no detrimental effect on bone mineralization.

### 3.3 Bone measurements

Table 4 shows that no significant (*p* > 0.01) difference was observed in the weight, length and diameter of bones among treatments. While there was a significant (*p* < 0.01) difference in all parameters between males and females, this difference was related to the weight because the weight of males was more than females. There was a significant (*p* < 0.01) interaction between treatments and sex. Results showed that males and females have different responses to the addition of SrR, CeO and their combination in the diet. As shown in Table 4, the length of the tibia in males was significantly (*p* < 0.01) different from females in all groups except in the group that was fed 600 mg/kg CeO, which showed an insignificant difference between females and males at the same time there is no difference with males in each of control, groups fed 450 mg/kg SrR and the combination.

### 3.4 Tibia mineral content in male chicken

Tibia minerals content in male birds showed significant (*p* < 0.01) differences among groups for Ca and Sr content, while phosphorus and magnesium content was not affected significantly (Table 5). The addition of SrR in the diet significantly (*p* < 0.01) reduced the content of Ca in both groups of SrR and in the group fed CeO (300 mg/kg feed) compared to the combination group. This may be due to the synergistic effects of both additives. On the other side, the opposite result was found in the SrR groups, the Sr content was higher significantly (*p* < 0.01) than in the other groups. This result is in agreement with the finding of Doberenz et al. (1969), which states that in small chicks Sr can replace Ca in the mineral of the growing bone. This may be due to the competition pattern between Ca and Sr to be absorbed in the intestine in which both are found in divalent charges and these two elements are chemically very close to each other thus the body absorbs Sr in the same way as Ca (Browning & Cowieson, 2013; Rajeshkumar et al., 2017; Wadkins & Peng, 1981). Also, Gad (2014) stated that non‐radioactive (stable Sr) is a chemical counterpart of Ca^2+^ and that Sr will replace Ca^2+^ in bones and other tissues with high Ca^2+^ concentrations. The higher level of Sr (0%, 0.12% or 0.24%) significantly reduced bone Ca content (34.7%) relative to controls (37.2%), suggesting that Sr replaced some of the Ca in the tibia (Shahnazari et al., 2006). A significant (*p* < 0.01) highest value of Ca content was found in the combinations group (SrR + CeO mixture) in the tibia compared to 450  and 900 mg/kg). This may be due to the synergistic effects of both additives. Insignificant effects were observed with the addition of CeO to the diet. The phosphorus and magnesium level in the tibia was not affected significantly. Similar outcomes were found by Browning and Cowieson (2015) in broiler chicken at 28 days old by adding 1200 mg Sr/kg to the diet.

**TABLE 5:** Effects of SrR, CeO and their mixture on the tibia mineral content in the male chicken broiler.

Treatments	Ca %	Sr %	P %	Mg %
Control	54.58 ± 0.311^abc^	2.09 ± 0.138^bc^	36.48 ± 0.868	3.97 ± 0.237
450 SrR	52.72 ± 0.530^c^	3.14 ± 0.146^a^	37.23 ± 0.339	4.79 ± 0.480
900 SrR	53.21 ± 0.605^bc^	3.03 ± 0.082^a^	36.74 ± 0.302	4.90 ± 0.244
300 CeO	53.35 ± 0.749^bc^	2.21 ± 0.189^bc^	36.56 ± 0.855	4.34 ± 0.413
600 CeO	54.90 ± 0.509^ab^	1.91 ± 0.052^c^	36.85 ± 0.133	3.93 ± 0.350
SrR + CeO	55.96 ± 0.406^a^	2.45 ± 0.133^b^	35.93 ± 0.471	3.28 ± 0.551
*p*‐value	0.0138	0.0001	0.08	0.083
CV	2.005	10.258	3.214	18.875

*Note*: Means ± SE. Abbreviations: CeO, cerium oxide; CV, coefficient of variance; F, female; M, male; SrR, strontium ranelate.

^a,..c.^ means with different letters within a column are significantly different (*p* < 0.01). *n* = 4. Treatment groups: Treatment 1: Control, Treatment 2: SrR (450), Treatment 3: SrR (900), Treatment 4: CeO (300), Treatment 5: CeO (600), Treatment 6: SrR + CeO (450 + 300) mg/kg. C

### 3.5 OC gene expression

**FIGURE 1:**
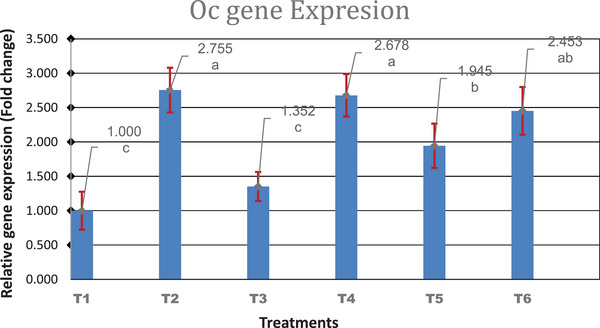
The expression level of osteocalcin (OC) in different groups. *n* = 4. Treatment groups: Treatment 1: Control, Treatment 2: SrR (450), Treatment 3: SrR (900), Treatment 4: CeO (300), Treatment 5: CeO (600), Treatment 6: SrR + CeO (450 + 300) mg/kg.

The effect of SrR and CeO and their combinations on the OC gene expression in males of broiler chickens is demonstrated in Figure 1. The results of real‐time PCR demonstrated that SrR and CeO and their combinations showed a significant (*p* < 0.01) impact on the expression of the OC gene, except for group‐fed SrR (900 mg/kg). The increased level of the OC gene indicates the generation of OC hormone in the osteoblasts. The altered function of bone cells, according to Pounds et al. (1991), could be related to changes in the calcium and adenosine 3′,5′‐cyclic monophosphate (cAMP) signalling systems in these cells. Also, Zhu et al. (2007) proved that SrR significantly enhanced the expression of OC in mice. Unexpectedly, the OC gene was more affected by adding a low level of SrR or CeO than a high level of both components.

### 3.6 ALP gene expression

**FIGURE 2:**
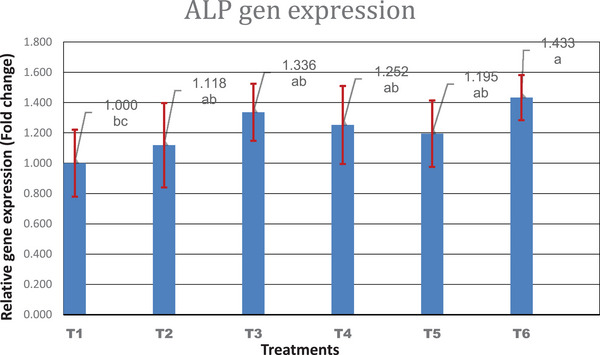
The expression level of alkaline phosphatase (ALP) in different groups. *n* = 4. Treatment groups: Treatment 1: Control, Treatment 2: SrR (450), Treatment 3: SrR (900), Treatment 4: CeO (300), Treatment 5: CeO (600), Treatment 6: SrR + CeO (450 + 300) mg/kg.

ALP is highly expressed in mineralized tissue cells and plays a critical role in the formation of hard tissue by increasing the inorganic phosphate local portion and facilitating mineralization while lowering extracellular pyrophosphate concentration, which is a mineral formation inhibitor (Vimalraj, 2020). Figure 2 shows the effect of SrR and CeO and their combinations on the ALP gene expression. The gene expression of ALP was increased significantly (*p* < 0.01) in the combination group (450 SrR + 300 CeO mg/kg feed) compared to the control group. Tsai et al. (2021) also reported that strontium‐substituted hydroxyapatite resulted in a significate impact on ALP activities.

## 4 CONCLUSION

The present study concludes that the addition of SrR, CeO and their combinations in the diet of broilers had no significant influence on live weight, BMC, BMD, BS, bone weight, length, and diameter. Moreover, the intake of combination minerals significantly increased BS in the females compared with the control group. There were significant interactions between treatments and sex for all parameters. Also, Sr can be replaced with Ca in the mineral content of the growing tibia. Higher gene expression of OC significantly was noticed in all treated groups compared to the control, except the group fed 900 mg/kg SrR. ALP gene expression was increased significantly in the combination group (450 SrR + 300 CeO mg/kg feed) compared to the control group. The SrR and CeO can be used as beneficial additives in the feed to improve the tibia quality of broilers.

## AUTHOR CONTRIBUTIONS

KAA is the project leader who is also the main supervisor to PSS who contributed to the idea, design and execution of the study. PSS performed the bone quality and measurements. KAA and PSS determined the expression level of genes. KAA and PSS were responsible for the statistical analysis. Both authors contributed equally to the write‐up of the final manuscript.

## CONFLICT OF INTEREST STATEMENT

The authors declare that there is no conflict of interest regarding the publication of this article. And all authors are in agreement with the content of the manuscript and submission in this journal.

## ETHICS STATEMENT

All applicable international, national, and institutional guidelines for the care and use of animals were followed. The protocol for animal care and the study was approved by the Animal Ethics Committee of the Animal Resources Department, College of Agriculture engineering sciences, Salahaddin University—Erbil, Iraq.

### PEER REVIEW

The peer review history for this article is available at https://publons.com/publon/10.1002/vms3.1190

## Data Availability

The data that support the findings of this study are available in the Supporting Information of this article.
